# Estimating diversity in networked ecological communities

**DOI:** 10.1093/biostatistics/kxaa015

**Published:** 2020-05-20

**Authors:** Amy D Willis, Bryan D Martin

**Affiliations:** Department of Biostatistics and Department of Statistics, University of Washington, Health Sciences Building, 1959 NE Pacific St, Seattle WA 98195, USA

**Keywords:** Diversity, Ecology, High throughput sequencing, Microbiome, Network

## Abstract

Comparing ecological communities across environmental gradients can be challenging, especially when the number of different taxonomic groups in the communities is large. In this setting, community-level summaries called *diversity indices* are widely used to detect changes in the community ecology. However, estimation of diversity indices has received relatively little attention from the statistical community. The most common estimates of diversity are the maximum likelihood estimates of the parameters of a multinomial model, even though the multinomial model implies strict assumptions about the sampling mechanism. In particular, the multinomial model prohibits ecological networks, where taxa positively and negatively co-occur. In this article, we leverage models from the compositional data literature that explicitly account for co-occurrence networks and use them to estimate diversity. Instead of proposing new diversity indices, we estimate popular diversity indices under these models. While the methodology is general, we illustrate the approach for the estimation of the Shannon, Simpson, Bray–Curtis, and Euclidean diversity indices. We contrast our method to multinomial, low-rank, and nonparametric methods for estimating diversity indices. Under simulation, we find that the greatest gains of the method are in strongly networked communities with many taxa. Therefore, to illustrate the method, we analyze the microbiome of seafloor basalts based on a 16S amplicon sequencing dataset with 1425 taxa and 12 communities.

## 1. Introduction

Microbial communities are composed of enormous numbers of different microbes, ranging from highly abundant taxa to rare taxa that are often unobserved. Data obtained from microbiome surveys often take the form of high-dimensional count data, generally with additional covariate information regarding the experimental conditions under which the communities were observed (Li, 2015). Detecting patterns in this data is challenging, partly because of its dimension. Analysis of *diversity* is a standard approach to summarizing and comparing high-dimensional community composition data in ecological studies and is ubiquitous in the microbiome literature (Callahan *and others*, 2016).

Consider a microbial community of $C$ taxonomic groups (taxa), which are present in relative abundances $z =(z_1, \ldots, z_C)$. Depending on the ecosystem under study, $C$ may be on the order of hundreds, but may also be in the tens of thousands or greater. An $\alpha$-diversity index $f: \mathbb{S}^{C-1} \to \mathbb{R}$ summarizes $z$, where $\mathbb{S}^d$ is the $d$-dimensional simplex. Similarly, $\beta$-diversity indices $g: \mathbb{S}^{C-1} \times \mathbb{S}^{C-1} \to \mathbb{R}$ summarize information from two communities. $\beta$-diversity indices summarize between-community structure, while $\alpha$-diversity indices summarize within-community structure. Specific examples are given in Section 2.

Despite the prevalence of $\alpha$- and $\beta$-diversity analyses in ecology, statistical methodology to estimate these functions is relatively underdeveloped. In particular, much of the existing literature focuses on estimating diversity under the assumption of observations drawn from a multinomial distribution with unknown probability vector $z$ (Miller, 1955; Zahl, 1977; Zhang and Zhou, 2010; Hsieh *and others*, 2016; Cao *and others*, 2019b). Fortunately, there exist sophisticated models for community composition data that permit a more flexible co-occurrence structure than that implied by the multinomial distribution. In this article, we utilize models from the compositional data literature that explicitly permit co-occurrence of taxa. The novelty of this article is in leveraging these models to estimate diversity indices, developing parametric and nonparametric variance estimates, and developing software implementing the method. Note that we do not propose novel diversity indices, but develop novel estimators of widely analyzed diversity indices.

In addition to incorporating network structure, the proposed method has a number of advantages over existing methods for diversity estimation. Most notably, while it is common in practice to estimate the diversity of each community individually, our method can pool information across multiple samples to estimate the diversity of the ecological communities from which the samples were drawn. Our methodology also permits a principled method for predicting diversity in communities that were not sampled. Our method achieves substantial improvements in estimation performance under simulation and is computationally feasible for modern microbiome datasets. The method is available as an R package at github.com/adw96/DivNet.

The manuscript is laid out as follows: Section 2 introduces methods for estimating $\alpha$- and $\beta$-diversity. In Section 3, we introduce our model for estimating diversity, and in Section 4, we discuss estimation of the model parameters and variance. Section 5 introduces a simulation study to evaluate the performance of the method (see also supplementary material available at *Biostatistics* online), and an example of the method is discussed in Section 6. We conclude with a discussion of the method, its limitations, and avenues for future research in Section 7.

## 2. Literature review: estimating $\alpha$- and $\beta$-diversity

Suppose that we have samples from $i=1,\ldots,n$ communities. Let $\mathcal{C}_i$ denote the set of all taxa in community $i$, and let $C_i = |\mathcal{C}_i|$ denote the number of taxa in the $i$th community. Let $\mathcal{C} = \cup_i \mathcal{C}_i$, $Q = |\mathcal{C}|$ and $q = 1, \ldots, Q$ index the taxa. Let $Z_{iq} \in [0,1]$ denote the (unknown) relative abundance of taxon $q$ in community $i$, noting that $\sum_{q=1}^Q Z_{iq} = 1$. (Note that while $Z_{iq}$ is an unknown parameter, in our model below we will treat it as a latent random variable, and so we use this notation throughout for consistency.) Associated with each community is a known vector of covariates $X_i \in \mathbb{R}^p$ where $p \geq 1$.

Suppose that from the $i$th community, $M_i$ individuals are observed and classified into the $Q$ taxonomic groups. Let $W_{iq}$ denote the number of times that taxon $q$ was observed in the sample from community $i$. Therefore, to estimate summary statistics associated with the communities, the information available on which to base estimation is $W \in \mathbb{R}^{n \times Q}$ and $X \in \mathbb{R}^{n \times p}$.

While members of an ecological community may differ in their levels of relatedness, to constrain the scope of this article we do not consider measures of diversity that are functions of taxonomy, such as Faith’s phylogenetic diversity (Faith, 1992), branch weighted phylogenetic diversity (McCoy and Matsen, 2013) or UniFrac (Lozupone and Knight, 2005).

### 2.1. $\alpha$-Diversity

There are a number of different $\alpha$-diversity indices that are widely used in the literature. This is because different indices reflect different features of communities. Two of the most common indices are the Shannon entropy (also called the Shannon index), and the Simpson index. While the diversity estimation framework that we will introduce is applicable to any $\alpha$-diversity index that is a function of taxon abundance (including any Hill number; Hill, 1973), we will focus on the Shannon and Simpson indices to illustrate our method.

#### 2.1.1. Shannon entropy

One of the most common $\alpha$-diversity indices is the Shannon entropy (Shannon, 1948). The Shannon index of community $i$ is defined as

(2.1) \begin{align*} \alpha_{i, Shannon} = -\sum_{q \in \mathcal{C}_i} Z_{iq} \log(Z_{iq}). \label{shannon_definition} \end{align*}

This index captures information about both the species richness (number of species) and the relative abundances of the species: as the number of species in the population increases, so does the Shannon index, and as the relative abundances diverge from a uniform distribution and become more unequal, the Shannon index decreases (for fixed $|\mathcal{C}_i|$, the entropy is maximized when $Z_{iq} = 1/C_i$ for all $q \in \mathcal{C}_i$).

Under the model $\mathbf{W}_i \sim {\rm Multinomial}(M_i, \mathbf{Z}_i)$, the maximum likelihood estimate (MLE) of $\alpha_{i, {\rm Shannon}}$ is $-\sum_{q \in \mathcal{C}_i} \frac{W_{iq}}{M_i} \log\left(\frac{W_{iq}}{M_i} \right),$ with the convention that if $W_{iq} = 0$, then $\frac{W_{iq}}{M_i} \log\left(\frac{W_{iq}}{M_i}\right) \equiv 0,$ since $\lim_{x \to 0} x \log x = 0.$ This estimate is almost ubiquitous in the ecological literature (Weiss *and others*, 2017). The multinomial MLE of Shannon diversity is often referred to as the plug-in estimate (Vu *and others*, 2007). The multinomial MLE is negatively biased by $\frac{|C_i|-1}{2M_i} + O(M_i^2)$ (Basharin, 1959), for which various corrections have been proposed, including adding $\frac{|C_i|-1}{2M_i}$ (the *Miller-Maddow* MLE correction; Miller, 1955), and jackknifing (Zahl, 1977).

Noting that unobserved (latent) taxa are often a substantial source of error in estimating the Shannon index, Chao and Shen (2003) proposed using the Good–Turing estimate of species richness and adjusting for the missing taxa, obtaining the estimate $ -\sum_{q \in \mathcal{C}_i} \frac{\hat{C}_i\hat{\pi}_{iq} \log(\hat{C}_i\hat{\pi}_{iq})}{1 - (1-\hat{C}_i\hat{\pi}_{iq})^n},$ where $\hat{\pi}_{iq} = W_{iq}/M_i$ and $\hat{C}_i = 1-\sum_{q} {1}_{\{W_{iq} = 1\}}/\sum_{q}W_{iq}$. Vu *and others* (2007) show that this estimator is consistent and converges with the optimal rate $O_P(1/\log (M_i)).$

More recently, Chao *and others* (2013) proposed to correct bias due to latent taxa by subsampling taxa and extrapolating from the sequentially smaller subsamples. The idea behind this method is to sample $m_1, m_2, \ldots, m_{k}$, microbes without replacement from the $M_i$ observed microbes. $k$ multinomial MLEs of the Shannon diversity are constructed based on each of the subsamples, and we call the $j$th estimate $\hat{\alpha}_i(m_j)$. The curve $\{m_j, \hat{\alpha}_i(m_j)\}_{j=1}^k$ is then constructed, along with an estimate of the slope of the curve. This curve is then extrapolated based on the estimated slopes to $m \rightarrow \infty$. The method is implemented in the R package iNEXT (Hsieh *and others*, 2016), with which we compare our method. We note that the taxa are subsampled independently to reflect the assumptions of the multinomial model. An alternative approach to adjusting for latent taxa originates in the compositional data analysis literature. To estimate the compositions $Z_{iq}$, Martín-Fernández *and others* (2003) propose replacing observed values of $W_{ij}$ that are exactly zero with 0.5, and so Cao *and others* (2019b) consider the resulting *zero-replace* $\alpha$-diversity estimator $-\sum_{q \in \mathcal{C}} \frac{W_{iq} \vee 0.5}{\sum_{r \in \mathcal{C}} W_{ir} \vee 0.5} \log\left(\frac{W_{iq} \vee 0.5}{\sum_{r \in \mathcal{C}} W_{ir} \vee 0.5}\right).$Cao *and others* (2019b) also extend this idea by fitting a Poisson-Multinomial model to $W$ via a regularization approach that penalizes the nuclear norm of $Z$, thereby obtaining a low-rank estimate of $Z$ that is close to the MLE under a Poisson-Multinomial model. No publicly available software implements the low-rank matrix method.

#### 2.1.2. Simpson index

Simpson (1949) defined the index now known as the *Simpson index*:

(2.2) \begin{align*} \alpha_{i, Si} = \sum_{q \in \mathcal{C}_i} Z_{iq}^2. \label{simpson_definition} \end{align*}

Similar to the Shannon index, the most common estimate of the Simpson index is the plug-in estimate $\hat{\alpha}_{i, Si,{\rm plug{\mbox -}in}} = \sum_{q \in \mathcal{C}_i} \left(\frac{W_{iq}}{M_i}\right)^2.$Zhang and Zhou (2010) demonstrated that under independent sampling from a multinomial distribution, $\frac{M_i}{M_i-1} \hat{\alpha}_{i, Si,{\rm plug{\mbox -}in}}$ is unbiased and asymptotically normally distributed. However, since $M_i$ generally exceeds 1000 in microbiome studies, the difference between the Zhang and Zhou (2010) and the plug-in estimate is negligible in our setting.

A number of approaches to estimating the Shannon index are also applicable to estimating the Simpson index. For example, Cao *and others* (2019b) investigate the performance of the zero-replace and low-rank approach to estimating the Simpson index. The extrapolation approach of Hsieh *and others* (2016) also applies to the Simpson index.

#### 2.1.3. $\alpha$-Diversity with covariates

With the exception of Cao *and others* (2019b), all of the estimates for $\alpha_i$ discussed above are only functions of the abundance vectors $\mathbf{W}_i$. Notably, none utilize the full abundance matrix $W$ nor the covariate matrix $X$. To address this, De’ath (2012) proposed a multinomial logistic regression approach to estimating the Shannon diversity. Advantages of this method include that diversity can be extrapolated, while disadvantages include a lack of publicly available software and no generative model for the species counts. More recently, Arbel *and others* (2016) proposed a nonparametric Bayesian model that exploits structure in $W$ as well as incorporating covariate information. Specifically, the model for the taxon counts $W$ given the taxon relative abundances $Z$ is nonparametric. The marginal prior distribution for $Z_i$ is the Griffiths–Engen–McCloskey distribution, and is a function of $X_i$. The method is computationally expensive, and at present, an implementation only exists for $p \leq 2$. We compare our method to the method of Arbel *and others* (2016) with respect to both estimation error and computation time. We also note the related method of Ren *and others* (2017), which also implements a nonparametric model for $W$ given $Z$ but with a marginal prior distribution for $Z_i$ given by a Gamma process. However, since it is unable to handle continuous covariates, we do not consider it further.

There exist many statistical models for species counts. However, most of these models do not model species relative abundances, and so cannot directly be used for estimating diversity indices that are functions of relative abundance. For example, the classical model of Dorazio and Royle (2005) models the presence and detection probabilities of species, but not species relative abundances (similar for Yamaura *and others*, 2011 but using presence data, rather than count data). Similarly, Hui *and others* (2015) (see also Letten *and others*, 2015) propose a latent variable model for species counts, and Pollock *and others* (2014) propose a model for species presence, but neither model estimates latent relative abundance. It has been previously noted (Gloor *and others*, 2016, 2017) that modeling relative abundance data with non-compositional models can lead to incorrect conclusions because the unit-sum constraint can alter the apparent direction of changes to the community. For example, if taxa 1, 2, and 3 exist in absolute abundance of, respectively, 100 units, 20 units, and 20 units before a treatment and 100 units, 40 units, and 20 units after a treatment, the relative abundance of taxa 1 and 3 have decreased, even though their absolute abundance was unchanged by the treatment. The model that we propose in Section 3 explicitly accounts for the compositional nature of microbiome data.

### 2.2. $\beta$-Diversity

Similar to $\alpha$-diversity, a large number of different $\beta$-diversity metrics exist, each highlighting different features of differences in communities. Legendre and Legendre (2012, Table 7.2) provide a list of 26 $\beta$-diversity metrics along with some discussion. However, in comparison to $\alpha$-diversity estimands, there exists almost no statistical literature on estimating $\beta$-diversity indices: estimating $\beta$-diversity indices is almost exclusively performed using plug-in estimators.

In general, small values of a $\beta$-diversity index indicate that the communities have similar compositions, while large values indicate that the relative abundances differ between communities, or that few taxa are shared by the communities. This interpretation holds for both the Bray–Curtis and Euclidean indices discussed below.

#### 2.2.1. Bray–Curtis dissimilarity

The (observed) Bray–Curtis index (Bray and Curtis, 1957) is defined as

(2.3) \begin{align*} \hat{\beta}_{ij, BC, {\rm plug{\mbox -}in}}=1-2\frac{\sum_{q \in \mathcal{C}_i \cup \mathcal{C}_j} \min(W_{iq}, W_{jq})}{M_i + M_j}.\label{bcdefinition} \end{align*}

While we have not found any discussion of the target estimand in the literature, (2.3) suggests that $\beta_{ij, BC}=1-\sum_{q \in \mathcal{C}} \min(Z_{iq}, Z_{jq})$ is the target estimand. Interestingly, in contrast to the other $\beta$-diversity indices discussed in the section, this estimate is not the MLE under a multinomial model.

While Arbel *and others* (2016) focused on estimating $\alpha$-diversity, because their method estimates the latent composition matrix $Z$, we also compare our proposed method to the estimate $1-\sum_{q \in \mathcal{C}} \min\left(\hat{Z}^{(\rm Arbel)}_{iq}, \hat{Z}^{(\rm Arbel)}_{jq}\right),$ where $\hat{Z}^{(\rm Arbel)}$ is the latent composition matrix estimate based on the procedure of Arbel *and others* (2016).

#### 2.2.2. Euclidean distance

Finally, we mention the Euclidean distance between the relative abundance vectors, $\beta_{ij, {\rm ED}}=\sqrt{\sum_{q \in \mathcal{C}}(Z_{iq} - Z_{jq})^2},$ whose plug-in estimate is $\hat{\beta}_{ij, {\rm ED}}=\sqrt{\sum_{q \in \mathcal{C}_i \cup \mathcal{C}_j} \left(\frac{W_{iq}}{M_i} - \frac{W_{jq}}{M_j}\right)^2}.$ We are not aware of any other estimates for the Euclidean distance between relative abundances in the literature, but we will also compare to the estimate $\hat{\beta}_{ij, {\rm ED, Arbel}}=\sqrt{\sum_{q \in \mathcal{C}}(\hat{Z}^{(\rm Arbel)}_{iq} - \hat{Z}^{(\rm Arbel)}_{jq})^2}.$

## 3. Estimating diversity in networked compositional data

Members of ecological communities interact, displaying repeatable patterns in many different environmental settings (Faust and Raes, 2012). For example, organisms may compete for resources, prey on each other, or cooperate in a symbiotic relationship. In the last decade, many methods have been developed to estimate the co-occurrence patterns of ecological communities, such as SparCC (Friedman and Alm, 2012) and SPIEC-EASI (Kurtz *and others*, 2015). We will refer to co-occurrence patterns as *ecological networks*. As we show under simulation, ecological networks can have substantial effects on estimates of diversity. Here, we propose an approach to estimating diversity in the presence of an ecological network.

### 3.1. Compositional data models

While the multinomial distribution is the canonical model for compositional data, the covariance between the number of observations in different categories is constrained to be negative. To deal with this issue, Aitchison (1982, 1986) developed the log-ratio model. This models the counts $W_{iq}$ as independent draws from a multinomial distribution,

(3.1) \begin{align*} p(W|Z) \propto \prod_{i = 1}^n \prod_{q = 1}^{Q} Z_{iq}^{W_{iq}}, \label{w_definition} \end{align*}

where $Z \in \mathbb{R}^{n\times {Q}}$ is a matrix-valued latent random variable that gives the underlying composition matrix for each of the communities: $\sum_{q = 1}^{Q} Z_{iq} = 1$ for all $i$. It then employs the log-ratio transformation by fixing a “baseline” taxon (taxon $D$) for comparison:

(3.2) \begin{align*} Y_{iq} = \phi(Z_{iq}) = \left\{ \log \left(\frac{Z_{iq}}{Z_{iD}} \right) \right\}_{q = 1, \ldots, D-1, D+1,\ldots {Q}}. \label{phi_definition} \end{align*}

Note that the log-ratio transformation $\phi: \mathbb{R}^Q \to \mathbb{R}^{Q-1}$ is invertible with inverse $\phi^{-1}$:

\begin{align*} Z_{iq} = \phi^{-1}(Y_{iq}) := \left\{ \begin{array}{ll} \frac{\exp(Y_{iq})}{\sum_{q \neq D}\exp(Y_{iq})+1} \hspace{1cm} q \neq D \notag\\ \frac{1}{\sum_{q \neq D}\exp(Y_{iq})+1} \hspace{1cm} q = D \end{array} \right\}. \notag \end{align*}

To permit flexible co-occurrence structures between the taxa, the log-ratios are modeled by a multivariate normal distribution:

(3.3) \begin{align*} f(\mathbf{Y}_i | \mu, \Sigma) \propto |\Sigma|^{-1/2} \exp \left\{ -\frac{1}{2} (\mathbf{Y}_i - \mu_i)^T \Sigma^{-1} (\mathbf{Y}_i - \mu_i)\right\}. \label{y_definition} \end{align*}

Finally, the mean of $\mathbf{Y}_i$ is linked to covariates via $\mu_i = X_i^T \gamma$, where $\gamma \in \mathbb{R}^{p \times (Q-1)}$. Under this model, $\gamma_{rq}$ gives the expected increase in $\log \left(\frac{Z_{i q}}{Z_{i D}} \right)$ for a one-unit increase in $X_{ir}$. For a discussion of the interpretation of this model on the scale of $Z_{iq}$, we refer the reader to Billheimer *and others* (2001).

This model does not impose that all communities with the same covariate vector $X_i$ have the same latent relative abundance vector $\mathbf{Z}_i$. However, under this model, the expectation of $\phi(\mathbf{Z}_i)$ is the same for all communities with the same covariate vector. Therefore, communities with the same environmental conditions are not constrained to have the same diversity under our model. We also note that this model is predicated on the assumption that the counts are conditionally independent given the covariate matrix $X \in \mathbb{R}^{n \times p}.$ Therefore, this model does not apply to spatially or temporally correlated data. We analyze the effect of temporal dependence in Section S2 of the supplementary material available at *Biostatistics* online.

### 3.2. Estimating diversity in the presence of a network

We propose using the log-ratio model to estimate $\alpha$-diversity and $\beta$-diversity. To our knowledge, this is the first proposal to estimate these diversity parameters under a model that explicitly models taxon–taxon co-occurrence structure. Let $\hat{\gamma}$ be an estimate of $\gamma$ under the log-ratio model. We take a frequentist approach to estimation, and discuss maximum likelihood estimators in Section 4.1 and penalized maximum likelihood estimators in Section 4.2.

Suppose that we wish to estimate the $\alpha$-diversity of a community with covariate vector $X_i \in \mathbb{R}^p$. Define $\hat{Y}_i = X^T_i \hat{\gamma},$ the expected value of the random variable $\mathbf{Y}_i$, and define $\hat{Z}_i = \phi^{-1}(\mathbf{Y}_i)$, the fitted value of the latent composition.

We then propose the following estimate of any $\alpha$-diversity index $f: \mathbb{S}^{C-1} \to \mathbb{R}$:

(3.4) \begin{align*} \hat{\alpha}_i = f(\hat{Z}_i). \end{align*}

More explicitly, $\hat{\alpha}_{i,{\rm Sh,proposed}} = -\sum_{q} \hat{Z}_{iq} \log \hat{Z}_{iq}$ and $ \hat{\alpha}_{i,{\rm Si,proposed}} = \sum_q (\hat{Z}_{iq})^2$ give our proposed estimates of the Shannon and Simpson indices. Similarly, for any $\beta$-diversity index $g: \mathbb{S}^{C-1} \times \mathbb{S}^{C-1} \to \mathbb{R}$, we propose

(3.5) \begin{align*} \hat{\beta}_{ij} = g(\hat{Z}_i, \hat{Z}_j), \end{align*}

such as $\hat{\beta}_{ij,{\rm BC, proposed}} = 1- \sum_q \min(\hat{Z}_{iq}, \hat{Z}_{jq})$ and $\hat{\beta}_{ij,{\rm ED, proposed}} = \sqrt{\sum_q (\hat{Z}_{iq} - \hat{Z}_{jq})^2}$ for the Bray–Curtis and Euclidean diversity indices. Note that if $\hat{\gamma}$ is the MLE of $\gamma$, then by invariance, the proposed estimates are the MLEs of the diversity indices.

This approach to diversity estimation has a number of key advantages not shared by other methods. Fundamentally, rather than describing a quantity associated with the sample (as is the case with plug-in estimates), the estimand is the diversity of the community from which the sample was drawn. This means that information is shared across all samples to obtain more precise and accurate estimates. Furthermore, we can use the model to estimate the diversity of communities for which ecosystem survey data are not available but for which covariate information exists. While these advantages are shared with the method of Arbel *and others* (2016), our method is fast and is available as an open-source R package with examples and tutorials illustrating its use.

## 4. Parameter estimation

### 4.1. Estimating model parameters

To estimate the parameter set $\eta = (\gamma, \Sigma)$, we take a frequentist approach via maximum likelihood. If $Y$ were known, our optimization problem would be to find

(4.1) \begin{align*} \hat{\eta} = argmax_{\eta}\sum_{i=1}^n \left[\log Pr(\mathbf{W}_i|\mathbf{Y}_i) + \log f(\mathbf{Y}_i|\eta) \right]\label{optprob}, \end{align*}

where

(4.2) \begin{align*} \log Pr(\mathbf{W}_i|\mathbf{Y}_i) &= \sum_{q \neq D} W_{iq}Y_{iq} - M_i \log\left(\sum_{q \neq D} \exp(Y_{im}) + 1\right) \end{align*}

and

(4.3) \begin{align*} \log f(\mathbf{Y}_i | \eta) &= - \frac{1}{2}\log(|\Sigma|) - \frac{1}{2} (\mathbf{Y}_i - \mu_i) \Sigma^{-1} (\mathbf{Y}_i -\mu_i). \end{align*}

Alas, since $Y$ is a latent random variable, we cannot directly optimize (4.1). Instead, we use the Expectation–Maximization (EM) algorithm (Dempster *and others*, 1977). The expected complete log-likelihood is

(4.4) \begin{align*} Q(\eta|\eta^{(t)}) &= -\frac{n}{2}\log(|\Sigma|) - \frac{1}{2}\sum_{i=1}^n \mathbb{E}_{Y|(W, \eta^{(t)})}[(\mathbf{Y}_i - \mu_i)^T\Sigma^{-1}(\mathbf{Y}_i - \mu_i)]. \label{qeqn} \end{align*}

To estimate this expectation numerically, we follow Xia *and others* (2013) and use the Metropolis–Hastings (MH) algorithm. Let $\left\{\mathbf{Y}_i^{(r)}\right\}_{r=1}^R$ be $R$ draws from the distribution of $\mathbf{Y}_i|\mathbf{W}_i,\eta^{(t)}$. Given these draws, we can approximate the expectation as follows:

(4.5) \begin{align*} \mathbb{E}_{Y|(W, \eta^{(t)})}[(\mathbf{Y}_i - \mu_i)^T\Sigma^{-1}(\mathbf{Y}_i - \mu_i)] &\approx \dfrac{1}{R}\sum_{r=1}^{R} (\mathbf{Y}_i^{(r)}-\mu_i^{(t)})^T(\Sigma^{(t)})^{\dagger}(\mathbf{Y}_i-\mu_i^{(t)}), \label{expectation_approximation} \end{align*}

where $\dagger$ is the generalized inverse.

To generate the $r$th draw from $f(\mathbf{Y}_i|\mathbf{W}_i,\eta^{(t)}),$ we simulate a proposal $\mathbf{Y}_i^{(*)}\sim \mathcal{N}_{Q-1}(\mathbf{Y}_i^{(r-1)},vI_{Q-1})$, where $v$ is a tuning parameter controlling the step size and $I_{Q-1}$ is the identity matrix of dimension $Q-1$. We then calculate the Metropolis acceptance ratio

$$r(\mathbf{Y}_i^{(*)}|\mathbf{Y}_i^{(r-1)}) = {\rm min} \left\{ 1,\dfrac{f(\mathbf{Y}_i^{(*)} | \mathbf{W}_i,\eta^{(t)})}{f(\mathbf{Y}_i^{(r-1)}|\mathbf{W}_i,\eta^{(t)})}\right\},$$

and simulate $u\sim \text{Uniform}(0,1)$. We set $\mathbf{Y}_i^{(r)} = \mathbf{Y}_i^{(*)}$ if $u\leq r(\mathbf{Y}_i^{(*)}|\mathbf{Y}_i^{(r-1)})$, otherwise, we set $\mathbf{Y}_i^{(r)}=\mathbf{Y}_i^{(r-1)}$. By initializing $\mathbf{Y}_i^{(0)} = \phi\left(\frac{\mathbf{W}_i}{M_i}\right)$, setting $v = 0.01$, and discarding the first $500$ draws, we observe convergence to the target distribution on a variety of microbiome datasets, and acceptance ratios ranging 30–40%.

Having obtained an estimate of the expectation in (4.4), we turn our attention to maximizing $Q(\eta|\eta^{(t-1)})$. Define $\eta^{(t)} = argmax_\eta Q(\eta|\eta^{(t-1)})$. Given our draws $\left\{\mathbf{Y}_i^{(r)}\right\}_{r=1}^R$ from $f(\mathbf{Y}_i|\mathbf{W}_i,\eta^{(t)})$, our M-step of the EM algorithm gives the following estimates:

(4.6) \begin{align*} \gamma^{(t+1)} &= \frac{1}{R}\sum_{r=1}^R (X^TX)^{\dagger} X^TY^{(r)},\\ \end{align*}

(4.7) \begin{align*} \mu_i^{(t+1)} &= X_i^T\gamma^{(t+1)}, \\ \end{align*}

(4.8) \begin{align*} \Sigma^{(t+1)} &= \dfrac{1}{nR}\sum_{r=1}^R \sum_{i=1}^n \left\{\mathbf{Y}_i^{(r)}-\mu_i^{(t)}\right\} \left\{\mathbf{Y}_i^{(r)}-\mu_i^{(t)}\right\} ^T, \label{sample_covariance} \end{align*}

where $X \in \mathbb{R}^{n \times p}$ and $Y^{(r)} = \left(\mathbf{Y}_1^{(r)}, \ldots, Y_n^{(r)}\right)^T \in \mathbb{R}^{n \times (Q-1)}$. Inspection of convergence diagnostics (such as trace plots) on a variety of datasets indicates that $R = 500$ and $\hat{\eta} = \eta^{(t)}$ for $t=6$ is generally sufficient to achieve stable estimates (see Section S4 of the supplementary material available at *Biostatistics* online). We run the MH algorithm to approximate the distribution of $\mathbf{Y}_i|\mathbf{W}_i,\eta^{(t)}$ in parallel over $i = 1, \ldots, n$ to reduce computation time. Our code is publicly available as an R package and can be found at github.com/adw96/DivNet.

### 4.2. Variance estimation

To test hypotheses about changes in diversity over environmental gradients, it is necessary to have accurate estimates of the variance of the diversity estimates. These variance estimates can then be used in hypothesis testing (e.g., using the method of Willis *and others* (2016)). We consider both parametric and nonparametric bootstrap approaches to estimating the variance of the diversity estimates produced by our model and evaluate them under simulation. For a given dataset $(W, X)$, let $\hat{\gamma}$ and $\hat{\Sigma}$ be the estimated values of $\gamma$ and $\Sigma$ estimated by the algorithm described in Section 4.1.

The parametric bootstrap approach to estimating ${\rm Var}(\hat{\alpha}_i)$ and ${\rm Var}(\hat{\beta}_{ij})$ for arbitrary diversity indices works as follows: $B$ datasets are simulated from the log-ratio model with $\mu = X\hat{\gamma}$ and $\Sigma = \hat{\Sigma}$. Then, for each of the $B$ simulated datasets, bootstrap estimates $\{(\hat{\gamma}^{(b)}, \hat{\Sigma}^{(b)})\}_{b=1}^B$ are obtained using the algorithm described in Section 4.1, and an estimate of the diversity index for community $i$ is obtained based on each simulated dataset (i.e., $\{\hat{\alpha}_i^{(b)}\}_{b=1}^B$). The parametric bootstrap estimate of ${\rm Var}(\hat{\alpha}_i)$ is then $\widehat{{\rm Var}}_b(\hat{\alpha}_i^{(b)})$, where $\widehat{{\rm Var}}(\cdot)$ is the sample variance. An estimate of the variance of any $\beta$-diversity index can be obtained in the same way.

We also consider a nonparametric bootstrap approach to estimating the variance of our estimates. We investigate the nonparametric bootstrap for completeness. To construct a nonparametric bootstrap estimate, we uniformly at random select with replacement $n_{\rm sub}$ elements from $\{1, \ldots, n\}$ to obtain a set which we call $\mathcal{B}$. We then estimate $(\hat{\gamma}^{(\mathcal{B})}, \hat{\Sigma}^{(\mathcal{B})})$ from $(W^{(\mathcal{B})}, X^{(\mathcal{B})})$, where $W^{(\mathcal{B})}$ and $X^{(\mathcal{B})}$ are the rows of $W$ and $X$ with row index in $\mathcal{B}$, and use $\{(\hat{\gamma}^{(\mathcal{B})}, \hat{\Sigma}^{(\mathcal{B})})\}$ estimates to obtain $\hat{\alpha}_i^{(\mathcal{B})}$. We repeat this process $B$ times to obtain a set of estimates $\{\hat{\alpha}_i^{(\mathcal{B}_b)}\}_{b=1}^B$ from which we calculate the nonparametric bootstrap estimate $\widehat{\rm Var}(\hat{\alpha}_i)= \widehat{\rm Var}_b (\hat{\alpha}_i^{(\mathcal{B}_b)})$ (and similarly for $\beta$-diversity).

The parameter $\Sigma$ drives the variance in the log-ratio model: as $||\Sigma||_\infty \to 0,$ the distribution of $W$ converges to a multinomial distribution. Therefore, the overdispersion of the log-ratio model relative to the multinomial model is driven by $\Sigma$. However, the number of taxa often greatly exceeds the number of communities obtained in microbiome surveys, and in this setting, $(\Sigma^{(t)})^\dagger$ may be a poor estimate of $\Sigma^{-1}$ in (4.5), even for large $t$. We therefore consider replacing $(\Sigma^{(t)})^\dagger$ in (4.5) with a regularized estimate obtained from the graphical lasso (Friedman *and others*, 2008; Witten *and others*, 2011). Following the popular microbial network estimation software SPIEC-EASI (Kurtz *and others*, 2015), we use stability selection to select the regularization parameter (Liu *and others*, 2010; Kurtz *and others*, 2015). We also consider replacing $(\Sigma^{(t)})^\dagger$ with the MLE restricted to the class of diagonal covariance matrices. Note that this approach to covariance estimation ignores variance attributable to inter-taxon interactions but allows for overdispersion relative to the multinomial due to within-taxon interactions.

We evaluate the performance of these 6 approaches to estimating the variance of diversity indices (two approaches to estimating the variance for each of three approaches to estimating the inverse covariance) under simulation. We design our simulation to mimic the dataset analyzed in Section 6, but with varying $Q$, the number of taxa and the size of the covariance matrix to be estimated. As is the case for the dataset of Section 6, we fix $p=2$, $n=12$, and set $X = (\mathbf{1}_n^T, (\mathbf{0}_{2n/3}, \mathbf{1}_{1n/3})^T)$. Note that our method can accommodate both discrete and continuous covariates, but we choose discrete covariates for all simulation studies to reflect the structure of the dataset analyzed in Section 6. Let $\mathcal{W}^Q$ be the columns of the count matrix $W$ of Section 6 corresponding to the $Q$ most common taxa over all samples. Let $\mathbf{Y}^Q_i = \phi(\mathcal{W}^Q_i) \in \mathbb{R}^{Q-1}$, and $Y^Q = [\mathbf{Y}_1^Q \cdots \mathbf{Y}_n^Q] \in \mathbb{R}^{n \times (Q-1)}$. We set $\gamma^Q = (X^TX)^{-1}X^TY^Q$ and $\Sigma^Q$ to be the covariance of the columns of $Y^Q - X\gamma^Q,$ and for each $Q$, we simulate data according to the log-ratio model with parameters $\gamma^Q$, $\Sigma^Q$ and $M_i = \sum_q W_{iq}$. Specifically, to simulate from the log-ratio model with parameters $(\gamma, \Sigma, X, M)$, we first simulate a matrix $Y \in \mathbb{R}^{n \times (Q-1)}$ with $i$th row $\mathbf{Y}_i \sim \mathcal{N}(X_i^T \gamma, \Sigma)$, then calculate the matrix $Z$ with $i$th row $\mathbf{Z}_i = \phi^{-1}(\mathbf{Y}_i)$ (see (3.2)), and finally simulate the matrix $W \in \mathbb{Z}^{n \times Q}$ with $\mathbf{W}_i \sim \text{Multinomial}(M_i, \mathbf{Z}_i)$. Noting that $n$ is small at $n=12$ (as is often the case for microbiome analyses), we choose $B = 3$ simulated datasets for the parametric bootstrap and $B = 3$ subsamples of size $n_{\rm sub} = 6$ for the nonparametric bootstrap approach, but to ensure that our simulation results are accurate we average over 25 simulation replicates.

**Fig. 1.**
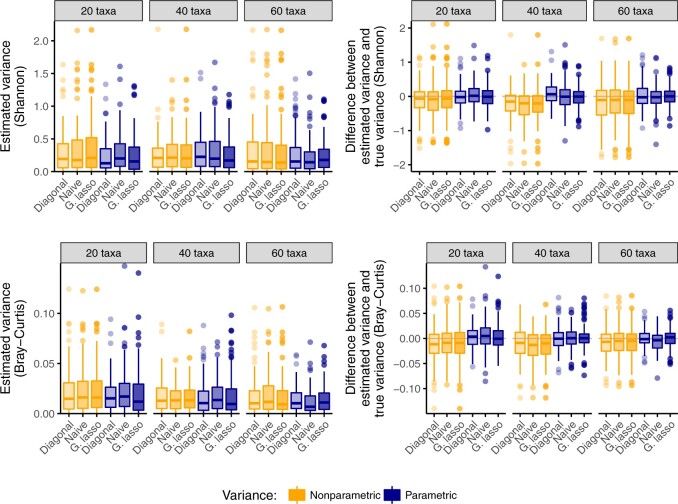
A comparison of nonparametric and parametric bootstrap approaches to estimating the variance of diversity estimates under a model that incorporates microbial co-occurrence patterns. The parametric bootstrap has lower variance than the nonparametric bootstrap (left panel), and the median difference with true variance close to zero (right panel). No approach to covariance estimation consistently outperforms other approaches.

We compare the estimated variance of the six methods in Figure 1 for a varying number of taxa $Q$. Only the variance of the Shannon index and Bray–Curtis index are shown, but similar patterns were observed for all indices. We observe that both parametric and nonparametric bootstrap variances are of similar magnitude, with parametric approaches generally having slightly lower median variance (left panels). In addition, to confirm that the estimated variance does not underestimate the true variance, we compare the difference between the estimated variance and the true variance for each method (right panels). The true variance of each method is estimated by repeatedly simulating data according to ($\gamma^Q$, $\Sigma^Q$, $M$), estimating the diversity index for each simulated dataset and each covariance estimate, and calculating the variance of the estimated indices. We observe that the median difference between the true variance and the stated variance is near zero for the parametric approaches, but negative for the nonparametric approaches, indicating that nonparametric approaches tend to underestimate the true variance. However, none of the three approaches to covariance estimation show substantial advantage over the others. This suggests that the primary driver of variance in estimating diversity in microbial communities is within-taxon interactions (the diagonal elements of $\Sigma$), rather than between-taxon interactions (the off-diagonal elements of $\Sigma$). Given these results, we select the naïve (generalized inverse of the sample covariance) approach to estimating $(\Sigma^{(t)})^{-1}$ as our default method. This approach is less computationally expensive than fitting the graphical lasso, while still permitting between-taxon interactions in the model. However, the functionality to estimate $\Sigma$ via a structured approach is implemented in our R package.

## 5. Simulation study

An investigation of the performance of our proposed method is available as supplementary material available at *Biostatistics* online. We investigated the performance of the method when data are generated according to the model described in Section 3.1 (Section S1 of the supplementary material available at *Biostatistics* online), when the data are generated according to the stochastically perturbed discrete-time Lotka–Volterra (LV) model of Fisher and Mehta (2014) (Section S2 of the supplementary material available at *Biostatistics* online), and when data are generated according to a nonlinear model on the log-ratio scale (Section S3 of the supplementary material available at *Biostatistics* online). In Section S1 of the supplementary material available at *Biostatistics* online, we investigated the effect of sample size (Section S1.1 of the supplementary material available at *Biostatistics* online), co-occurrence structure (Section S1.2 of the supplementary material available at *Biostatistics* online), and number of taxa (Section S1.3 of the supplementary material available at *Biostatistics* online). In Section S2 of the supplementary material available at *Biostatistics* online, we investigated the effect of number of taxa and number of time points. In Section S3 of the supplementary material available at *Biostatistics* online, we investigated both a quadratic and an exponential trend and varied the degree of curvature for each. We found that the proposed method strongly outperforms competitors when data are generated according to the model described in Section 3.1. When data are generated according to the stochastically perturbed discrete-time LV model, its performance suffers, especially when there are a large number of taxa and a small number of time points. However, estimation is relatively robust to nonlinear trends. We refer the reader to supplementary material available at *Biostatistics* online for details on the data generating processes and our results.

## 6. Data analysis: seafloor microbial diversity

Because of its coarse nature as a community-level summary, diversity analyses are especially relevant to studies of novel ecological communities. Lee *and others* (2015) collected and analyzed microbial communities living on seafloor rocks on the Dorado Outcrop, an area of exposed basalt on the East Pacific Rise. Hydrothermal vents such as the Dorado Outcrop inform our understanding of microbe–mineral interactions in the subsurface. Samples were collected from the seafloor rock, including glassy, altered basalts (“glassy,” $n = 4$) and highly altered basalts (“altered,” $n = 8$). Analysis of the microbial communities on these rocks revealed 1425 distinct microbial taxa in glassy and altered basalts after filtering for low quality sequences (see Lee *and others* (2015) and Lee (2018) for details surrounding sequencing and construction of the abundance table). Here, we investigate if the community-level structure differs between the different rock types.

**Fig. 2.**
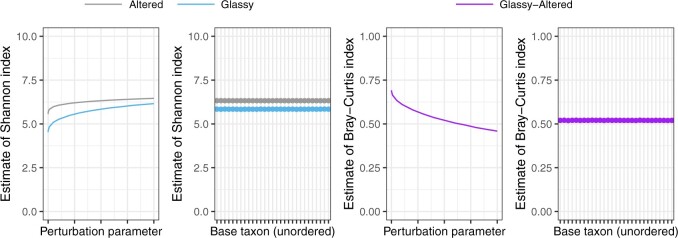
The log-ratio model described in Section 3 can only be fit to data with a minimum abundance greater than zero. Abundance data for microbiome studies are generally sparse, and 42% of the observed abundances of the Lee *and others* (2015) dataset are zero. For this reason, it is common to add a perturbation offset $\rho$ to the observed abundance table before fitting the log-ratio model. Here, we see that the estimated diversity does depend on the choice of $\rho$.

We investigate 30 choices for the $Q$th taxon, whose abundance will be the denominator in the calculated log-ratios. Since $\frac{\partial}{\partial y}\log(x/y) = -1/y$ is smallest in absolute value for large $y$, we investigate the effect of setting $Q$ to be a high abundance taxon. In particular, there were 86 amplicon sequence variants (ASVs) that were present in all samples, and so we uniformly at random select 10 ASVs from this collection of 86 ASVs, and compare the estimates of diversity obtained by setting each of these 10 taxa as the denominator taxon. We contrast these estimates with those obtained from ranging $Q$ across the 10 most abundant taxa over all samples. We also compare 10 randomly selected taxa. The estimated Shannon, Simpson, Jaccard, and Euclidean diversities are shown in Figure 2 (2nd and 4th panels), indicating that, in practice, the diversity estimates are almost invariant to the choice of base taxon. We select $Q$ to be ASV 2, (a Nitrospirae of order Nitrospirales), which was the most abundant taxon that was observed in every sample.

In contrast to the stability of diversity estimates with varying $D$, we find that the effect of perturbing the zero counts can be substantial (Figure 2, 1st and 3rd panels). As noted previously (Martín-Fernández *and others*, 2003; Cao *and others*, 2019a,b, $W_{ij}$ is commonly zero for microbiome data, because many taxa do not occur in every sample (42% of the entries of our abundance table are zero). However $f(x, y) = \log(x/y)$ is only defined for $x,y>0$, and so it is common to perturb the original abundance data $W$ by adding a perturbation factor $\rho \in (0,1)$ to create a new abundance table $W_{ij}^{(\rho)} = W_{ij} + \rho$, and the modeling the perturbed data $W^{(\rho)}$. In Figure 2, we observe sizeable changes in the diversity estimates when varying $p$ close to zero (at most 26%, $-$50%, $-$24%, and $-$31% changes in Shannon, Simpson, Bray-Curtis, and Euclidean estimates for $\rho=0.001$ compared to $\rho=0.5$), but smaller changes when $\rho$ is increased from 0.5 to 1 (at most 5%, $-$24%, $-$12%, and $-$13% changes for $\rho=0.5$ to $\rho=1$). We therefore follow Cao *and others* (2019b) and choose $p=0.5$ as the perturbation parameter for the remainder of our analysis.

Throughout this article, we have argued that the multinomial model is misspecified for microbiome data. To investigate this claim for the dataset of Lee *and others* (2015), we fit the log-ratio model and calculate the eigenvalues of $\hat{\Sigma}.$ Since the multinomial model is the limit of the model described in Section 3.1 as $\Sigma \rightarrow 0$, and the largest eigenvalue of $\hat{\Sigma}$ for this dataset is 422.87, this is strong evidence that the multinomial model is misspecified for this dataset.

**Fig. 3.**
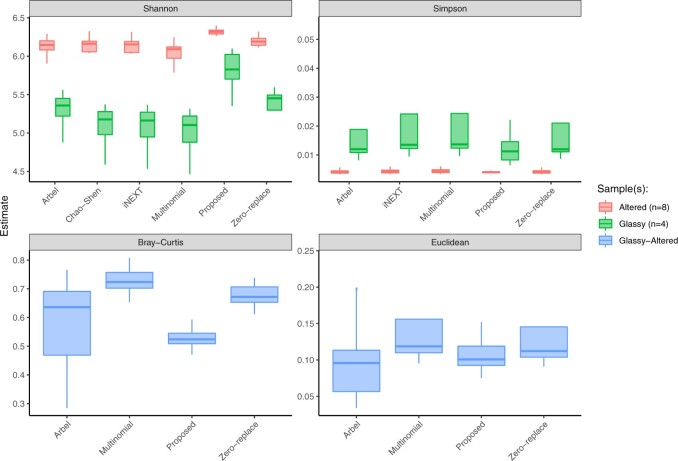
Lee *and others* (2015) collected and analyzed microbial communities living on different types of seafloor basalts on the Dorado Outcrop. Here, we compare a variety of estimators for four diversity indices. 25% and 75% quantiles are shown.

Finally, we compare our estimates to the estimates obtained from other methods. Interval estimates are shown in Figure 3. The proposed method was fit in mode tuning = “careful” the method of Arbel *and others* (2016) was run for 500 iterations, and convergence was confirmed via trace plots; and the method of Chao and Shen (2003) was run with the default $k=40$ and 50 bootstrap resamples. Code for repeating the analysis is available at github.com/adw96/DivNet_supplementary.

While most methods produce similar estimates, we note a number of advantages of our proposal. Firstly, any diversity index that is a function of relative abundance can be estimated using our method, unlike the methods of Hsieh *and others* (2016) and Chao and Shen (2003). Secondly, our interval estimates are more symmetric around the median of the bootstrapped estimates compared to other estimates. Thirdly, while this analysis only included two covariates, our method can handle multiple covariates.

## 7. Discussion

Despite substantial evidence that strong co-occurrence networks exist in microbial communities, and a growing body of literature concerned with estimating co-occurrence networks, no methods that explicitly incorporate co-occurrence networks into diversity estimation currently exist. Here we propose a new method, called DivNet, to fill this gap. DivNet is highly accurate when the log-ratio model is correctly specified, including when there are a large number of taxa. DivNet can be used to model count data arising from direct observations, flow cytometry, or high throughput sequencing technologies such as 16S amplicon sequencing. It is available as an open-source R package via github.com/adw96/DivNet.

By leveraging information from multiple samples, DivNet can estimate the relative abundance of a taxon in a community where it was not observed. However, a limitation of DivNet is that it does not estimate the number of taxa that were missing in all samples. Therefore, when there are a large number of latent taxa, DivNet may miss the effects of these low abundance taxa. This weakness is shared by the estimators of Arbel *and others* (2016) and Cao *and others* (2019b), while the estimators of Hsieh *and others* (2016) and Chao and Shen (2003) adjust for missing taxa (but are only applicable to $\alpha$-diversity). However, the latter two estimators cannot handle covariates nor repeated samples, which contribute to the performance of our method. In the situation when no replicates or covariates are available, there are a large number of latent taxa, and $\beta$-diversity is not of interest, a practitioner may prefer these methods.

Under simulation, we demonstrated that DivNet performs favorably when data are generated independently and identically from a distribution where the taxa co-occur on the log-ratio relative abundance scale. This generally holds even when the set of covariates is misspecified, such as when there is an exponential trend on the log-ratio scale, but a linear or quadratic model is fit. We also found that the performance of DivNet suffers when data are generated according to a LV model. Under this model, the abundances of taxa are temporally correlated, and the co-occurrence network acts on the absolute, not relative, abundant scale. We found that for short LV-simulated time series with many taxa, other estimators may outperform DivNet. We note that other violations of conditional independence are likely to adversely affect DivNet’s performance, including spatial correlation. We encourage caution when applying DivNet to count data where observations are not independent. In practice, since the data generating process is generally not known, we recommend that the user contrast a number of different estimators before drawing conclusions about diversity.

We also note that it is common for ecologists to be interested in the ordering of diversity indices rather than their absolute values. We are not aware of a data analysis where the ordering of diversity across a covariate has been altered by the choice to estimate diversity using DivNet. However, because the log-ratio model is overdispersed compared to a multinomial model, the standard errors of DivNet are larger than the standard errors for the MLE of a multinomial model, reflecting the additional uncertainty captured by the model.

We suggest four avenues for further research that would build upon our proposed method. The first is to construct an estimator under the log-ratio model that estimates the number of missing taxa. However, this would require a principled approach to estimating the ecological network of a taxon that was not observed in any sample. A second avenue for research is to impose some structure (e.g., sparsity) on the relative abundance parameter $\gamma$, whose dimension is large when there are a large number of taxa. Thirdly, since diversity indices that incorporate relative abundance and phylogenetic information are commonly used by ecologists, extending the method to incorporate phylogeny is an open problem. Finally, a generalization of the method that relaxes the assumption of independence to account for correlation between observations (e.g., due to spatial or temporal dependence) is yet to be developed, and is likely to outperform DivNet when observations are correlated.

The method described in this manuscript is available at github.com/adw96/DivNet. Code to reproduce the simulations, figures, and data analysis is available at github.com/adw96/DivNet_supplementary.

## Supplementary Material

kxaa015_Supplementary_Data
