# Supplementary material for: Estimating diversity in networked ecological communities
Source: Biostatistics. 2020 May 20;23(1):207–22. doi: 10.1093/biostatistics/kxaa015 (PMC8759443; doi:10.1093/biostatistics/kxaa015)
Supplement: kxaa015_Supplementary_Data [file kxaa015_supplementary_data.pdf]

Biostatistics (2020), **0**, 0, *pp.* 1–17
doi:10.1093/biostatistics/r3-supplementary

Supplementary material: Estimating diversity in networked ecological communities

AMY D. WILLIS AND BRYAN D. MARTIN

In this document we investigate the performance of the proposed method under simulation. All code to reproduce the simulations is available at `https://github.com/adw96/DivNet_supplementary`.

S1. Simulation study: Correct model specification

In this section we compare the performance of our proposal to estimates obtained from other methods, simulating W from the data generating procedure described in Section 4.2. We simulate from this model by specifying $Z \in \mathbb{R}^{n \times Q}$ and $M \in \mathbb{R}^n$. Since the `DivNet` estimator was developed for this data generating process, we expect `DivNet` to outperform other methods in these simulations.

To construct the matrix of latent relative abundances Z, we can specify $\mathbf{Y}_i$ for all $i = 1, \ldots, n$ then set $Z_{iq} = \phi^{-1}(Y_{iq})$. Since our model specifies that $\mathbf{Y}_i \sim \mathcal{N}(\mu_i, \Sigma)$, we therefore can specify μ_i and Σ and simulate $\mathbf{Y}_i$ for all i to obtain Z. We set $X = (\mathbf{1}_n^T, (\mathbf{0}_{n/2}, \mathbf{1}_{n/2})^T)$, and simulate a $2 \times (Q-1)$-dimensional matrix γ with independent $\mathcal{N}(0, \sigma_\gamma^2)$ entries ($\gamma_{rq} \overset{iid}{\sim} N(0, \sigma_\gamma^2)$ for $r = 1, 2$ and $q = 1, \ldots, Q-1$), and set $\mu = X\gamma \in \mathbb{R}^{n \times (Q-1)}$. μ_i is then the ith row of μ. To constrain the number of simulations we choose $\sigma_\gamma^2 = 1$ throughout. Note that the true relative abundance vector for sample i is $\mathbf{Z}_i = \phi^{-1}(\mu_i)$, and the true diversity estimands are calculated based on $\mathbf{Z}_i$.

© The Author 2020. Published by Oxford University Press. All rights reserved. For permissions, please e-mail: journals.permissions@oup.com

To construct Σ, we simulate a matrix $A \in \mathbb{R}^{(Q-1)\times(Q-1)}$ with elements drawn from a Uniform$(-1, 1)$ distribution, and construct a diagonal matrix D with diagonal elements forming an arithmetic sequence of length Q beginning at σ_{max} and decreasing to a minimum of σ_{min}. We then set $\Sigma = A^T DA$. Specifying Σ in this way allows us to compare the performance of our method across various Σ, but also to control the strength of network relationships between taxa via the parameter σ_{max}.

For each of the 4 diversity indices that we consider (Shannon, Simpson, Bray-Curtis, and Euclidean), we obtain an estimate under the multinomial model and using the proposed estimation procedure. The procedure of Arbel et al. (2016) can be applied when $p = 2$, and so we set $p = 2$ and choose $X = (\mathbf{1}_n^T, (\mathbf{0}_{n/2}, \mathbf{1}_{n/2})^T)$ for all simulations (noting that our method can accommodate both discrete and continuous covariates). The `R` package `iNEXT` (Hsieh et al. 2016) applies to estimating Shannon and Simpson α-diversity indices, but not to estimating β-diversity indices. Note that many of the Shannon diversity estimates are almost identical to the Multinomial MLE for large values of M_i (M_i is commonly 10^5 or greater in microbiome studies), including the estimates of Chao & Shen (2003) and Miller (1955), and for this reason we do not compare them here. For the same reason we also do not show the Simpson diversity estimate of Zhang & Zhou (2010). We use the `simulator` (Bien 2016) to manage the simulation study.

In all of the simulations that follow, we run the proposed method with `tuning = "fast"`, which runs 6 iterations of the EM algorithm and 500 Metropolis-Hastings steps, of which 250 were discarded as burn-in. We chose these values because they gave a reasonable balance of precision and speed (see Section S4 for justification). Note that the default behavior of the software in mode `tuning = "careful"` is 10 EM steps and 1000 MC steps (500 discarded as burn-in), and this is the mode that we recommend for data analysis. We ran `iNEXT` with the default behavior of 40 knots and 50 bootstrap iterations. While the default behavior of Arbel et al. (2016) is 10 MC iterations (80% are discarded as burn-in), we chose 500 MC iterations for the simulations

that follow (80% are discarded as burn-in), since we found that this was sufficient to achieve convergence of the Monte Carlo chain when $n = 40$ and $Q = 100$ (the largest values of n and Q that we investigated under simulation). We performed a sensitivity analysis and found that increasing the number of MC iterations in the method of Arbel et al. (2016) did not reduce its MSE. See Section S4 for a comparison and discussion of the computation times of `DivNet` and the method of Arbel et al. (2016).

Throughout this section we evaluate α-diversity estimates using the mean squared error (MSE) over all samples. The MSE of the kth simulation is $MSE_\alpha(\hat{D}^{(k)}) = \frac{1}{n}\sum_{i=1}^{n}(\hat{D}_i^{(k)} - D_i)^2$ where i indexes the estimates for each of the n samples. We similarly evaluate the β-diversity estimates: $MSE_\beta(\hat{D}^{(k)}) = \frac{1}{n(n-1)/2}\sum_{i<j}(\hat{D}_{ij}^{(k)} - D_{ij})^2$.

S1.1 *Estimation error decreases with sample size*

In this section we set $Q = 20$, $\sigma_{min} = 0.01$, $\sigma_{max} = 5$, and $M_i = 10^5$ for all i, and perform $K = 200$ simulations for each choice of n. The performance of the proposed method for estimating diversity when data are simulated under this model is illustrated in Figure 1. For all values of n and all diversity estimands, the 25%, 50%, and 75% quantiles of $\{MSE(\hat{D}^{(k)})\}_k$ are uniformly lower for our proposed method compared to all other methods. The improvement is especially pronounced for the β-diversity indices.

We find that the estimation error decreases as the sample size n increases for the proposed method and the method of Arbel et al. (2016), but not for the Multinomial MLE and the `iNEXT` method (Figure 1). This is unsurprising, since neither the plug-in nor `iNEXT` estimates use information contained in the covariate matrix X in their estimates of diversity. Therefore, the additional information afforded by larger values of n is not leveraged by the plug-in nor `iNEXT` estimates, even when experimental replicates are available.

The results shown in Figure 1 are based on fitting our model with $t = 6$ EM steps and $r = 500$

Fig. 1. A comparison of the error of different estimators for α- and β-diversity for microbial communities when the taxa are networked. When the network is ignored by the estimation procedure (e.g., Chao & Shen (2003), Hsieh et al. (2016) and the widely used "plug-in" estimate (multinomial MLE)), the error in estimating diversity can be substantial. The proposed estimation procedure, which specifically accounts for networks, outperforms other estimates for any sample size n. The distribution of mean squared errors (MSEs) is shown for 200 simulated datasets. In this simulation, there are $M = 10^5$ microbes observed per sample, $p = 2$ predictors and $Q = 20$ taxa.

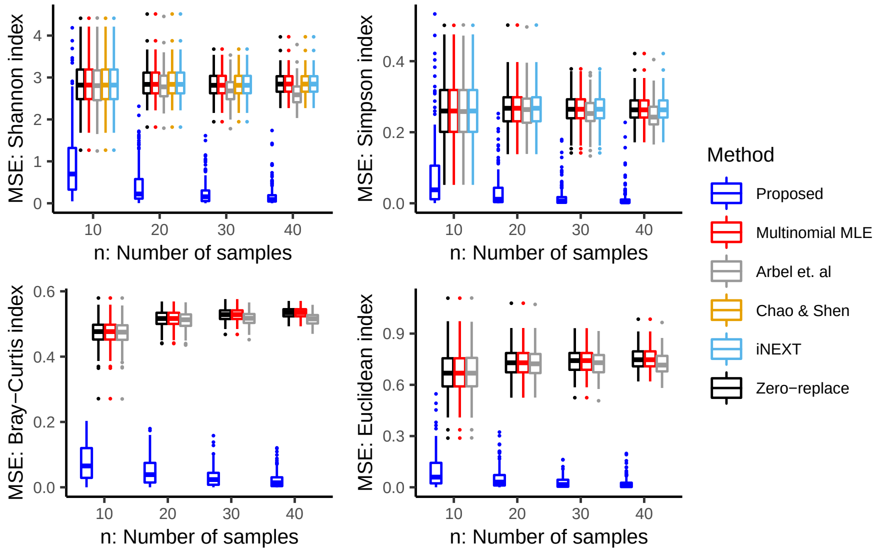

Fig. 2. A comparison of the computing time of different estimators of diversity indices. Our parallelized EM-MH algorithm for estimation under a network model is competitive with closed-form estimates, and is substantially faster to compute than the rarefaction-extrapolation approach of `iNEXT` (Hsieh et al. 2016) and the nonparametric Bayesian approach of Arbel et al. (2016). The computation time of the 200 datasets used to produce Figures 1 (left) and the computation time of the 100 datasets used to produce Figures 4 (right) are shown.

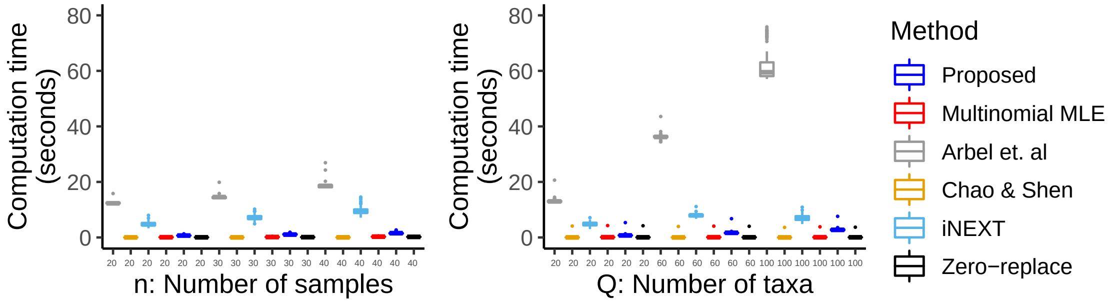

MH draws per EM step. For these choices, we show computation time in Figure 2. Fitting our model with $t = 6$ EM steps and $r = 500$ is more computationally expensive than calculating the plug-in estimate, but less computationally expensive than fitting the model of Arbel et al. (2016) (with 500 Monte Carlo iterations) or using the package `iNEXT` (with 40 knots and 50 bootstrap resamples). We note that our implementation leverages the `R` package `parallel` (R Core Team 2017) for parallelizing the MH algorithm employed at each E-step of the EM algorithm. See Section S4 for a full comparison of speed and MSE with varying numbers of E-steps for the proposed method and Monte Carlo steps for Arbel et al. (2016).

S1.2 *Estimation error is stable across networked communities*

We now investigate the effect of varying the co-occurrence structure. To vary the covariance structure in a systematic way, we vary σ_{max}, the largest eigenvalue of Σ. We now set $n = 20$, $Q = 20$, $\sigma_{min} = 0.01$, $M_i = 10^5$ for all i, and perform $K = 100$ iterations for each choice of σ_{max}. The results are shown in Figure 3. We see that estimating the diversity in microbial communities with strong occurrence structures is more challenging than estimating diversity in communities with co-occurrence structures similar to that of a multinomial model. However, the proposed method has lower MSE than all other methods that were investigated. Additionally, even when microbial abundances are simulated under a model with strong co-occurrence relationships, the proposed method can estimate the diversity with small MSE (Figure 3). In contrast, the estimation error increases as the co-occurrence relationships strengthen for all other methods. Co-occurrence relationships in microbial ecosystems are well documented (Faust & Raes 2012), indicating that a diversity estimation method tailored to networked ecosystems is of practical utility.

Fig. 3. Diversity estimates that incorporate network structure dominate estimators that do not incorporate network structure in the presence of a strong co-occurrence network. However, network-based estimates perform well even when there is a very weak network structure. As $\sigma_{max} \to 0$, the network model converges to the multinomial model. However, we see that the proposed network model performs equally as well or better than estimates based on the multinomial model for all choices of σ_{max}. This appears to be the case for estimating both α- and β-diversity.

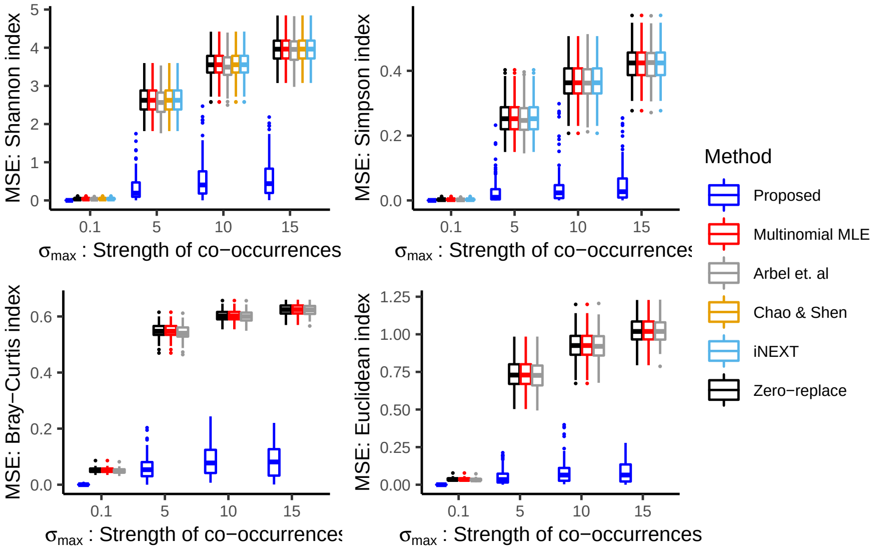

S1.3 *Estimation error is stable across large communities*

Finally, since microbial communities often contain many taxa, we wish to confirm the performance of our estimator in large communities. We set $n = 20$, $\sigma_{max} = 5$, $\sigma_{min} = 0.01$, $M_i = 10^5$, and perform $K = 100$ simulations. In Figure 4, we see that the estimation error for the proposed method remains low even as the size of the community increases, while all other methods have increasing estimation error. In particular, we note that this is true even though the simulated communities are networked ($\sigma_{max} = 5$), and the number of taxa exceeds the number of samples ($n = 20$). We therefore conclude that the procedure is appropriate for analyzing the diversity of microbial communities.

Fig. 4. Diversity estimates that incorporate network structure dominate estimators that do not incorporate network structure over communities of any size. While most estimators have increasing error for larger communities, the proposed estimator's error does not. In this simulation, we set $n = 20$ and $\sigma_{max} = 5$.

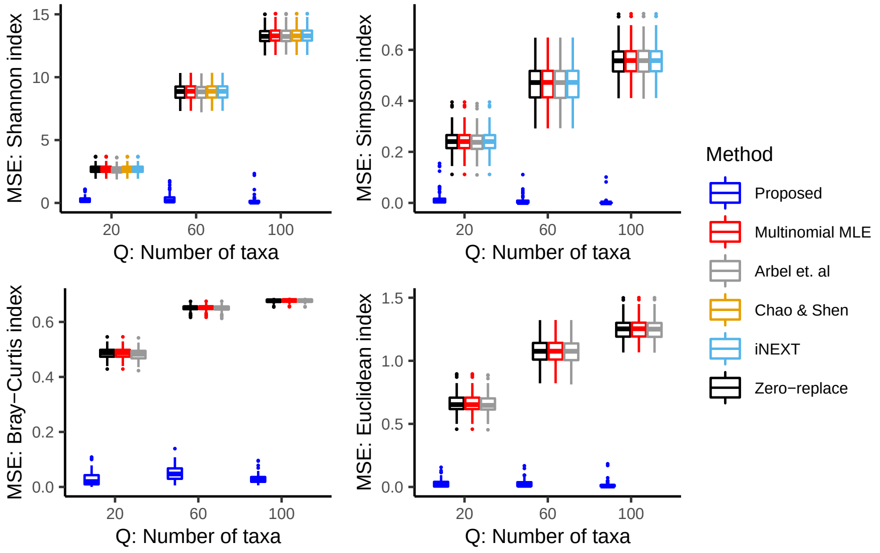

S2. Simulation study: Temporally correlated data

In Section S1 we investigated the performance of `DivNet` when counts are simulated according to the model described in Section 3.1 of the paper. Since `DivNet` was developed for this data generating process, this amounts to the most favorable case for estimation. We now investigate the performance of `DivNet` when data is generated according to the stochastically-perturbed discrete-time Lotka-Volterra model of Fisher & Mehta (2014). The discrete-time Lotka-Volterra model is a population dynamics model that states that the absolute abundance of taxon q at time $t + \delta t$, which we call $V_q(t + \delta t)$, is proportional to the absolute abundance of taxon q at time t, and each taxon's abundance is affected by a matrix of "interaction coefficients" $\{c_{ij}\}$ that model the effect that taxon j has on the abundance of taxon i. Fisher & Mehta (2014) generalized this model by introducing an additional stochastic perturbation $\eta_q(t)$ to reflect noise in the measurements. The dynamics of the stochastically-perturbed discrete-time Lotka-Volterra

model are given by the equation

$$V_q(t+\delta t) = \eta_q(t) V_q(t) \exp\left(\delta t \sum_{q'=1}^{Q} c_{qq'}(V_{q'}(t) - \tilde{V}_{q'})\right), \tag{S2.1}$$

where $\tilde{V}_{q'}$ is the steady-state absolute abundance of taxon q'.

Let $W(t) = \{W_1(t), \ldots, W_q(t)\}$ denote the counts observed from taxa $1, \ldots, q$ at time t, and let $Z(t) = \{Z_1(t), \ldots, Z_q(t)\}$ denote the latent relative abundances of the taxa at time t. In this simulation we will simulate data from (S2.1) and evaluate the error of our `DivNet` estimator for the steady-state Shannon diversity $\alpha_{Shannon}(\tilde{V})$.

We simulated count data $W(t) \sim Multinomial(10^5, Z(t))$, where the latent relative abundance of taxon q is $Z_q(t) = V_q(t)/\sum_{q'=1}^{Q} V_{q'}(t)$, and the true absolute abundances $\{V_q(t)\}$ are simulated according to the stochastically-perturbed discrete-time Lotka-Volterra model. We consider $t = 0, 1, \ldots, T$ and $q = 1, \ldots, Q$ for varying T and Q. The distributions of the parameters of the model are the same as those investigated by Fisher & Mehta (2014), which were based on a longitudinal study of the human gut microbiome. Specifically, $log(\tilde{V}_q) \overset{iid}{\sim} \mathcal{N}(0, \sigma = 0.1)$, $\eta_q(t) \overset{iid}{\sim} \mathcal{N}(0, \sigma = 0.01)$, and the interaction matrices c were constructed as follows:

1. Initialize the matrix c with all zero interactions
2. Assign diagonal entries according to $c_{qq} \overset{iid}{\sim} Uniform(-1.9/\tilde{V}_q, -0.1/\tilde{V}_q)$.
3. Choose a random off-diagonal position $\{k, l\}$. Draw

   $$c_{kl} \sim |c_{kk}| \times ((2 \times Beta(shape = 1, scale = 1)) - 1).$$

   Then, simulate $\{V(1), \ldots, V(T)\}$ according to the current interaction matrix c. If any $V_q(t)$ exceeds $2^{31} - 1$, repeat Step 3 until a stable c is found.
4. Repeat Step (3) until 10 interactions ($k \neq l$) have been specified.

A timeseries of length 60 for 20 taxa is shown in Figure 5 (top panel). Solid lines indicate the taxa abundances, dashed lines indicate the steady-state abundances, and each colour represents a

single taxon. Note that the distribution of $c_{qq'}$'s as following a Beta distribution was not described in the manuscript of Fisher & Mehta (2014); this information is contained in Line 646 of Fisher & Mehta (2014)'s Supporting Information Code S1, a Mathematica script.

We consider the MSE for estimating $\alpha_{Shannon}(\tilde{V})$ of a single community sampled longitudinally. The data that each estimator has available is a time series $V_q(t)$ of $T+1$ observations $(t = 0, 1, \ldots, T)$ of Q taxa.

We contrast the performance of four estimators of $\alpha_{Shannon}(\tilde{V})$. In this section, the plug-in estimator is $\frac{1}{T}\sum_{t=0}^{T} \hat{\alpha}_{plugin}(W(t))$, where $\hat{\alpha}_{plugin}(W(t))$ is the plug-in Shannon diversity estimator based on the counts observed at time t. Stated differently, the plug-in estimator of the steady-state Shannon diversity is the mean of the plug-in Shannon diversities at each of the timepoints $t = 0, 1, \ldots, T$. Similarly, in this section the Chao-Shen estimator is the mean of the Chao-Shen estimators at each time t, and the `iNEXT` estimator is the mean of the `iNEXT` estimators at each time t. The `DivNet` estimator takes $\left(W = (W(0), \ldots, W(t))^T, X = \mathbf{1}_T\right)$. In this simulation, since there are no covariates, we do not fit the estimator of Arbel et al. (2016).

The `DivNet` estimator is misspecified in two fundamental ways under this data generating process. Firstly, the data generating process is a perturbed Lotka-Volterra model, in which species interactions occur on the absolute abundance scale. Furthermore, the relative abundances are temporally correlated, while the `DivNet` estimator is built for the setting where observations are independent.

Our simulations show that the performance of the Chao-Shen, `iNEXT` and plug-in estimators for estimating $\alpha_{Shannon}(\tilde{V})$ are similar across all simulations (Figure 5), but that the performance of `DivNet` varies with Q and T. In the lower left panel, we show the distribution of squared errors when $Q = \{15, 30, 45\}$ and $T = 80$. We see that all estimators' estimation errors are negatively affected by larger Q, which is consistent with the view that more diverse communities are more challenging to model. `DivNet` has the lowest 1st, 2nd and 3rd quartiles of squared error when

$Q = 15$. In contrast, the median squared error is approximately the same for all methods when $Q = 30$, but the squared error of `DivNet` has more variance than its competitors. `DivNet` performs poorest out of all methods when $Q = 45$, with the highest median squared error. We conclude that the performance of `DivNet` depends on the number of taxa when data is generated according to the perturbed Lotka-Volterra model. `DivNet` may have advantages when the community has few taxa, but the advantages diminish for larger numbers of taxa and other methods may be preferable for very diverse communities. We contrast this with `DivNet`'s strong performance with an increasing number of taxa when the model is correctly specified (Figure 4).

In the lower right panel of Figure 5 we show the distribution of squared errors when $Q = 20$ and $T = \{20, 60, 100\}$. The estimation error of all estimators improves with T, which is consistent with our intuition that longer time series lead to lower estimation error. `DivNet` has lower estimation error than competitors when $T = 60$ and $T = 100$, but not when $T = 20$. This suggests that users should be cautious when applying `DivNet` to short, highly correlated timeseries data, but can expect superior performance to other methods over longer time series.

We conclude that the performance of `DivNet` is negatively affected by model misspecification, and the degree to which it is negatively affected depends on the dimension of the data, both with respect to number of taxa and number of observations in a longitudinal sampling setting.

S3. Simulation study: Non-linear trends

We now consider the performance of our proposed method when taxa respond non-linearly to covariates. We explore two scenarios: one in which the log-ratio-transformed relative abundance of one taxon follows a quadratic function and another in which the relative abundance of one taxon follows an exponential function. In this simulation we fix $n = 20$ and $Q = 100$, and vary the degree of non-linearity in the relative abundance of one taxon.

In the first simulation, we set $\mu_{i1} = 1 - \gamma X_i(X_i - 10)$ and $\mu_{iq} = 0$ for all $q \neq 1$: this amounts

Fig. 5. The relative advantages of the proposed method depend on the number of taxa in the community and number of longitudinal observations when data is generated according to the model of Fisher & Mehta (2014). We show an example timeseries of the absolute abundance of taxa in the top panel (hyperparameters are described in the text). Solid lines indicate the taxa abundances, dashed lines indicate the steady-state abundances, and each colour represents a single taxon. The lower panels show the squared error in estimating the steady-state Shannon diversity of a community of Q taxa observed over T timepoints. In the lower left panel T is fixed at $T = 80$, and in the lower right panel Q is fixed at $Q = 20$. `DivNet` performs best relative to other methods when Q is small and T is high.

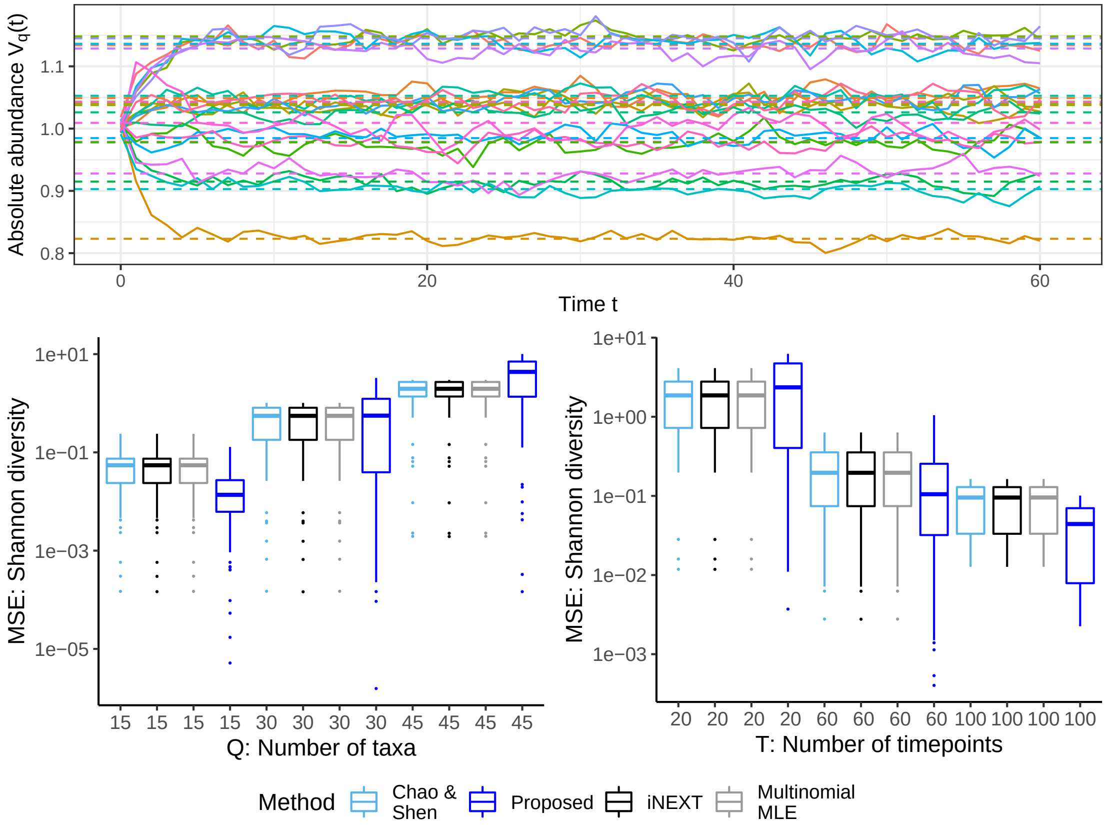

to a non-monotone quadratic function for the expected log-ratio-transformed relative abundance of taxon 1, and an equal expected relative abundance of the remaining taxa. We show how this model changes the expected community composition and its diversity in Figure 6. The upper left panel shows μ_{i1} as a function of X_i: this is the scale on which the model is quadratic. The upper middle panel shows Z_{i1} as a function of X_i: this shows how the relative abundance of taxon 1 increases from 0.02 to a maximum of approximately 0.25. The upper right panel shows how this

change in the abundance of taxon 1 affects the Shannon diversity of the whole community. We vary γ across $\{0.125, 0.25, 0.50\}$ to control the degree of non-linearity in the log-ratio-transformed relative abundance of taxon 1. This generative model for the Z_i's was chosen arbitrarily to give an example of a non-linear quadratic function in which the relative abundance of one taxon increases from low ($\sim 2\%$) to high ($\sim 25\%$) over the range of the covariates in such a way that the Shannon diversity of the community also displays a non-linear trend.

In the second simulation, we set $\mu_{i1} = 3 + 0.25e^{\gamma X_i}$ and $\mu_{iq} = 0$ for all $q \neq 1$: this amounts to a monotone non-linear function for the log-ratio-transformed relative abundance of taxon 1, and an equal relative abundance of the remaining taxa. We vary γ across $\{0.125, 0.25, 0.32\}$ to control the degree of non-linearity in the log-ratio-transformed relative abundance of taxon 1. This generative model for the Z_i's was chosen arbitrarily to give an example of a non-linear function in which the relative abundance of one taxon varies from low ($\sim 8\%$) to high ($\sim 18\%$) over the range of the covariates such that the Shannon diversity also displays a non-linear trend. The log-ratio-transformed relative abundance of taxon 1, relative abundance of taxon 1 and Shannon diversity of the community are shown in the lower panels of Figure 6.

For both simulations, we simulate $W_i \sim Multinomial(10^5, Z_i)$, where $Z_{iq} = \phi^{-1}(Y_{iq})$ and $\mathbf{Y}_i \sim \mathcal{N}(\mu_i, \Sigma)$. $\mu_{i1} = 1 - \gamma X_i(X_i - 10)$ for simulation 1 and $\mu_{i1} = 3 + 0.25e^{\gamma X_i}$ for simulation 2, $\mu_{iq} = 0$ for all $q \neq 1$ in both simulations, and in both simulations, $X_i = \left\{0, 1 \times \frac{10}{19}, 2 \times \frac{10}{19}, \ldots, 10\right\}$ such that $n = 20$. In each simulation, Σ was drawn randomly as described in Section S1.1 with $\sigma_{min} = 0.01$ and $\sigma_{max} = 5$.

In Figure 7 we illustrate the performance of all methods with respect to MSE for estimating the true Shannon diversity of each community. We investigate the performance of two options for our proposed method: one where it is fit with design matrix $\mathbf{X} = \left(\mathbf{1}_n, \tilde{X}, \tilde{X}^2\right)$ where $\tilde{X} = (X_1, \ldots, X_n)^T$, and another where it is fit with design matrix $\mathbf{X} = \left(\mathbf{1}_n, \tilde{X}\right)$. We call the former "Proposed (Quadratic)" and the latter "Proposed (Linear)". We compare these options with the

plug-in (Multinomial) estimate, the Chao-Shen estimate, the `iNEXT` estimate and the Arbel et al. (2016) estimate. The current implementation of Arbel et al. (2016) only accommodates $p \leqslant 2$, and so we investigate its performance with the covariates set to $\mathbf{X} = \left(\mathbf{1}_n, \tilde{X}\right)$ and to $\mathbf{X} = \left(\mathbf{1}_n, \tilde{X}^2\right)$, which we call “Arbel et al. (Linear)” and “Arbel et al. (Quadratic)”, respectively.

We see that `DivNet` performs well with respect to MSE for estimating the Shannon index even in the presence of non-linear trends (Figure 7). In the presence of a quadratic trend (Figure 7, upper panel), the proposed method with a quadratic trend generally has the lowest median squared error, though the proposed method fit with a linear trend has the lowest MSE in the low curvature quadratic trend case ($\gamma = 0.125$). This is most likely because the low curvature case can be well approximated by a linear trend, and fewer parameters need to be estimated in the linear model. However, when the model is misspecified (a linear model is fit even though the trend is quadratic), the error distribution can outperform ($\gamma = 0.125$), underperform ($\gamma = 0.25$) or be comparable to other methods ($\gamma = 0.5$). This underscores the risks of omitting a relevant covariate from the model.

In the presence of an exponential trend, both the linear and quadratic proposed models outperform other methods with respect to MSE (Figure 7, lower panel). In both the exponential and quadratic trend cases, we observe that the proposed method’s advantages over other methods diminish as the amount of curvature increases.

This simulation illustrates that while the performance of the proposed method is generally good even when the true data generating process is non-linear in the covariates, the specific shape of the trend log-ratio relative abundance can affect the performance of the method. Therefore, we recommend that the user exercises caution when the trend log-ratio relative abundances displays high curvature across the range of covariates of interest. While the true relative abundances are unknown, the sample log-ratio relative abundances can be easily plotted if the number of taxa is not large. If the number of taxa is large, we recommend inspecting the log-ratio relative abun-

Fig. 6. We investigate the performance of the method under model misspecification. We investigate quadratic (top row) and exponential (bottom row) trends. In this figure we show the log-ratio-transformed relative abundance of taxon 1 (left panels), relative abundance of taxon 1 (middle panels) and Shannon diversity of the community (right panels). The parameter γ controls the degree of curvature, with greater values of γ corresponding to greater curvature in the log-ratio-transformed relative abundance of taxon 1. $n = 20$ and $Q = 100$ in this simulation.

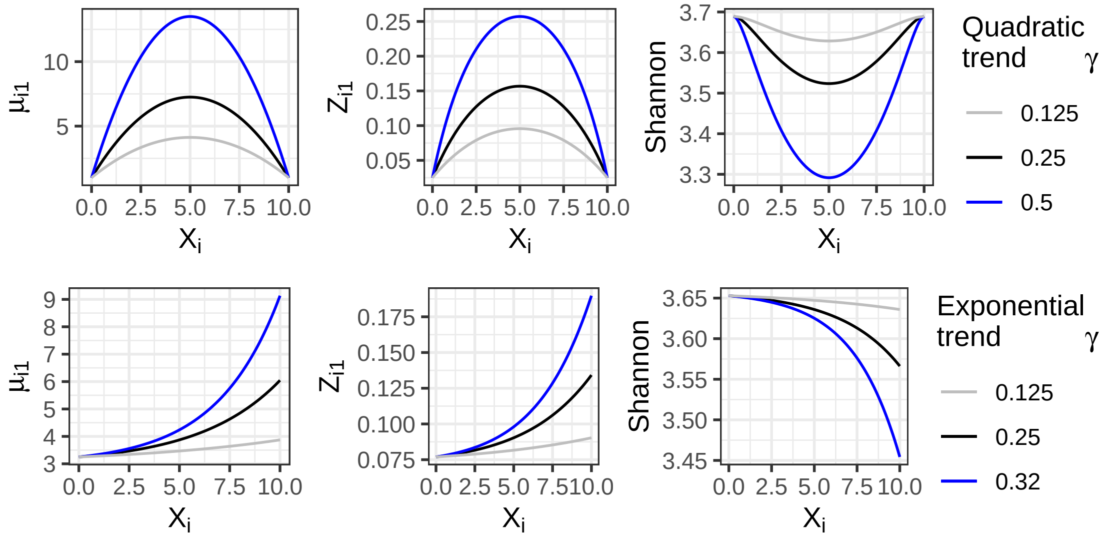

dances for the most abundant taxa. An alternative approach is to fit both linear and quadratic models to confirm that the obtained scientific results are robust to the selected model.

S4. Computation time of iterative procedures

The computational burden of our method increases with the number of iterations of the EM algorithm. Similarly, the computational burden of method of Arbel et al. (2016) increases with the number of Monte Carlo iterations. To compare these two methods with respect to speed and accuracy, we simulate data according to the same data generating procedure as in Section S1 with $n = 40$, $Q = 100$, and $\sigma_{max} = 5$ for each of 10 simulations. We compare the MSE and computation time of the method of Arbel et al. (2016) with 250, 500, 1000, and 2000 Monte Carlo iterations, with 80% of iterations discarded as burn-in (80% is the default burn-in fraction for this method). We compare these results with our method with 6, 10 and 20 iterations in the

Fig. 7. The estimation error for estimating the Shannon diversity depends on the data generating process. The proposed method strongly outperforms competitors when curvature in the data generating process is low (small values of γ), but the methods are more comparable as γ increases. This is true for both quadratic (top) and exponential (bottom) trends in the log-ratio transformed relative abundances. $n = 20$ and $Q = 100$ in this simulation.

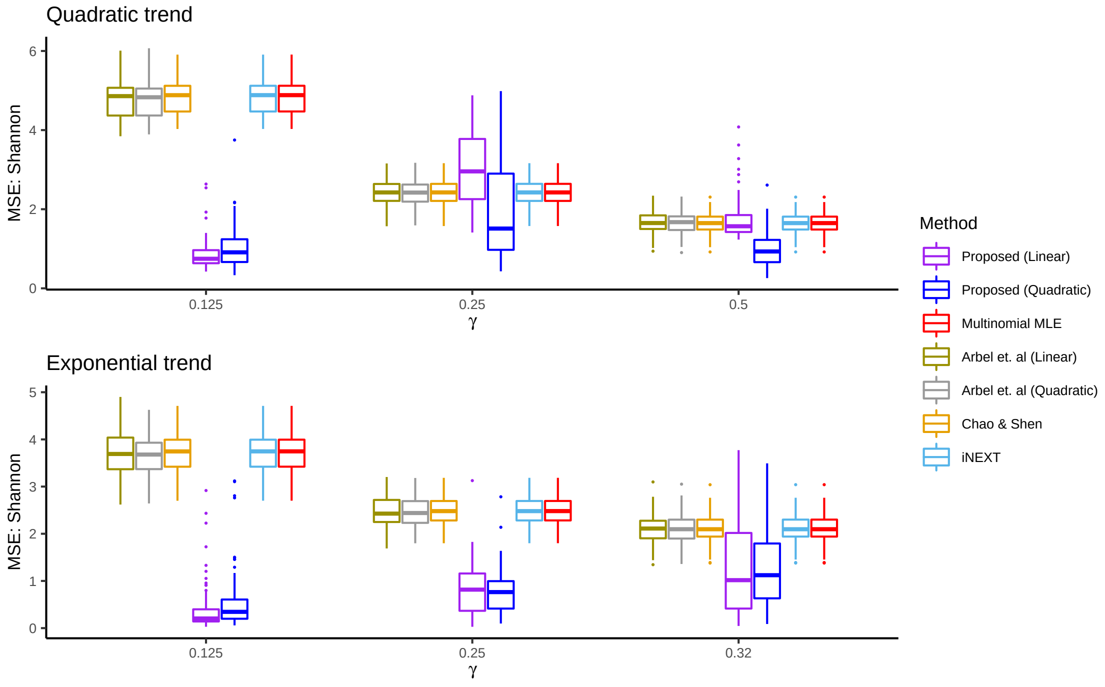

EM algorithm.

We see that for this data generating process, 250 iterations is not sufficient for the Bayesian sampler of Arbel et al. (2016) to converge, but 500 iterations is sufficient (Figure 8). However, more than 500 iterations does not improve the MSE. Our method converges after 6 EM iterations, which runs on a dataset of this size in a median of 6 seconds, compared to 89 seconds for the method of Arbel et al. (2016) with 500 MC iterations. Therefore even though both procedures are iterative, we believe that running 500 MC iterations for Arbel et al. (2016) and 6 iterations for the proposed method allows for a fair comparison of computation time and MSE in Figures 1 and 2.

Fig. 8. Both the procedure of Arbel et al. (2016) and the proposed method are iterative procedures. We simulate 10 datasets from the data generating procedure described in Section S1 with $n = 40$, $Q = 100$, and $\sigma_{max} = 5$ and compare the proposed method with a varying number of EM iterations with the method of Arbel et al. (2016) with a varying number of MC iterations. We see that the method of Arbel et al. (2016) converges after 500 MC iterations, and our proposed method converges after 6 EM iterations.

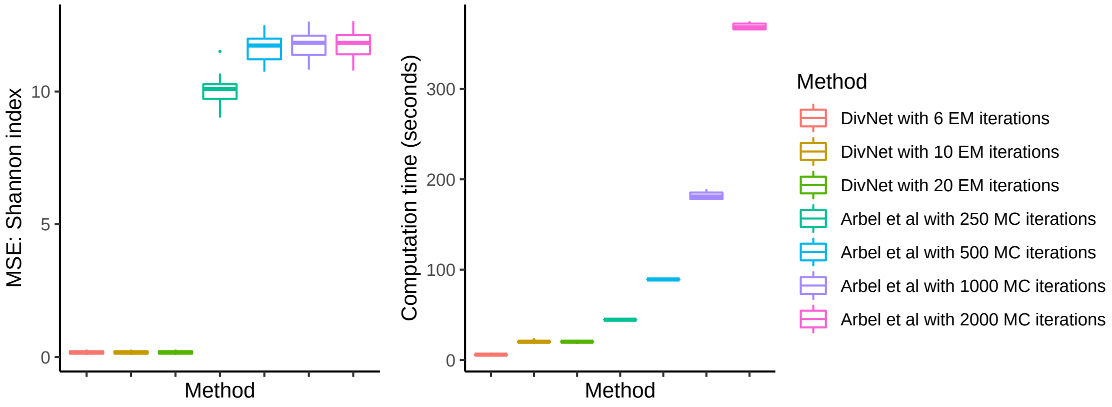

References

Arbel, J., Mengersen, K. & Rousseau, J. (2016), 'Bayesian nonparametric dependent model for partially replicated data: The influence of fuel spills on species diversity', *The Annals of Applied Statistics* **10**(3), 1496–1516.

Bien, J. (2016), 'The Simulator: An Engine to Streamline Simulations', *arXiv preprint arXiv:1607.00021* .

Chao, A. & Shen, T.-J. (2003), 'Nonparametric estimation of Shannon{\textquoteright}s index of diversity when there are unseen species in sample', *Environmental and Ecological Statistics* **10**(4), 429–443.

Faust, K. & Raes, J. (2012), 'Microbial interactions: from networks to models', *Nature Reviews Microbiology* **10**(8), 538.

Fisher, C. K. & Mehta, P. (2014), 'Identifying keystone species in the human gut microbiome from metagenomic timeseries using sparse linear regression', *PloS one* **9**(7), e102451.

Hsieh, T. C., Ma, K. H. & Chao, A. (2016), 'iNEXT: an R package for rarefaction and extrapolation of species diversity (Hill numbers)', *Methods in Ecology and Evolution* **7**(12), 1451–1456.

Miller, G. A. (1955), 'Note on the bias of information estimates', *Information theory in psychology: Problems and methods* **2**(95), 100.

R Core Team (2017), *R: A Language and Environment for Statistical Computing*, R Foundation for Statistical Computing, Vienna, Austria.

Zhang, Z. & Zhou, J. (2010), 'Re-parameterization of multinomial distributions and diversity indices', *Journal of Statistical Planning and Inference* **140**(7), 1731–1738.

[*Received TODO 1, 2010; revised TODO 1, 2010 TODO*]
